# Structured Online Support to Inform and Assist Antidepressant Deprescribing in Primary Care (WiserAD Tool): Protocol for a Pragmatic Randomized Controlled Trial

**DOI:** 10.2196/81858

**Published:** 2026-08-07

**Authors:** Catherine Kaylor-Hughes, Amy Coe, Patty Chondros, Konstancja Densley, Susan Fletcher, Mary-Lou Chatterton, Daniel Hoyer, Timothy Chen, Chee Ng, Derelie Mangin, Tony Kendrick, Zoe Allnutt, Jane Gunn AO

**Affiliations:** 1 Department of General Practice and Primary Care, University of Melbourne, Medical Building, Melbourne, 3010, Australia, 61 3 90355511; 2 School of Public Health and Preventive Medicine, Monash University, Melbourne, Australia; 3 Department of Biochemistry & Pharmacology, University of Melbourne, Melbourne, Australia; 4 Florey Institute of Neuroscience and Mental Health, Victoria, Australia; 5 Department of Molecular Medicine, Scripps Research Institute, La Jolla, CA, United States; 6 Sydney Pharmacy School, University of Sydney, Sydney, Australia; 7 Department of Psychiatry, University of Melbourne, Melbourne, Australia; 8 Department of Family Medicine, McMaster University, Hamilton, ON, Canada; 9 Department of General Practice, University of Otago, Christchurch, New Zealand; 10 School of Primary Care, Population Sciences and Medical Education, University of Southampton, Southampton, United Kingdom

**Keywords:** deprescribing, antidepressant, tapering, depression, anxiety, primary care, general practice, online, support tool, GP, RCT, Australia

## Abstract

**Background:**

The use of antidepressants is increasing globally. Despite their obvious benefits, ongoing use of these medications is often not properly monitored or deprescribed when a person returns to better mental health. In addition, providing prescriptions to those who do not have clinical depression leads to personal and societal cost burdens.

**Objective:**

This trial aims to assess the clinical effectiveness and cost-effectiveness of an online support tool designed to help patients with mild to no symptoms of depression and their general practitioners manage the careful and appropriate tapering and cessation of antidepressants at 6 months, and compare the effectiveness to that of usual care.

**Methods:**

This stratified, single-blind, parallel, 2-arm, superiority randomized controlled trial includes Australian primary care patients (aged 18-75 years) with mild to no symptoms of depression who have been on antidepressant medication for longer than 12 months. After obtaining informed consent, 340 eligible patients will be randomized in a 1:1 ratio to an active intervention arm and an attention control arm, with stratification by general practice or state of residence, if recruited via social media. Those in the active intervention arm will be asked to reduce their antidepressant use with the aid of a clinically guided online support tool (WiserAD), while those in the attention control arm will continue to receive usual care. Participants in both arms will be provided with information about antidepressants through the Beyond Blue website and followed up at 3, 6, 12, and 18 months to record antidepressant use, depression and anxiety symptom severity, quality of life, and health economic data. An intention-to-treat analysis will determine the clinical effectiveness of the online tool compared with usual care. The primary outcome is the between-arm difference in the proportion of participants who successfully cease medication use at 6 months and have mild or absent depressive symptoms. Cost-consequence and cost-utility analyses will be used to determine the cost-effectiveness of the intervention and its impact on quality of life, and comparisons will be made with usual care.

**Results:**

The study was funded by the National Health and Medical Research Council in 2019. At submission of this manuscript in July 2025, 310 participants have been randomized, and recruitment ongoing. The target number of 340 randomized participants was achieved in January 2026. Trial outcomes will be reported in peer-reviewed journals in Febraury 2027.

**Conclusions:**

The WiserAD online support tool assists patients and their general practitioners with deprescribing and may lead to successful cessation of antidepressant medication, resulting in enhanced quality of life and cost savings over the longer term.

## Introduction

### Background

Antidepressant medications have substantially improved the health and well-being of many people [1], and their success in enabling those experiencing an episode of depression to retain their quality of life has undoubtedly led to their widespread use globally. However, antidepressant use has led to a high and unnecessary economic burden on the health care system and patients through overprescribing. The usual “course length” for antidepressants in an episode of depression is a maximum of 12 months [2-4], and overprescribing occurs most often when these medications are not appropriately discontinued (termed “legacy prescribing”), when they are no longer clinically necessary, or where they are of no therapeutic use (ie, in the absence of a diagnosed or current indication for use). This represents a real opportunity to improve prescribing. Data indicate that 46% of those who start taking an antidepressant continue taking it for longer than the indicated period, and 61% of those currently prescribed antidepressants fall into this group, with the median length of prescription being 5 years [5].

Such inappropriate medicine use [6,7] is not aligned with current clinical guidelines [2,4] and is a clinical challenge for health care providers globally. The most recent figures from the Organisation for Economic Co-operation and Development show that Australia now has the fourth highest number of users of antidepressants per capita, with the defined daily dose (per 1000 people) increasing nearly 3-fold since 2000 [8,9], and 87% of these medications are prescribed through general practitioners (GPs) [10]. This is largely due to an excess of long-term users [11,12] rather than a higher number of people being newly diagnosed with major depressive disorder (MDD) or other disorders for which antidepressants are prescribed (eg, anxiety) [13,14]. Several factors drive the inappropriate use of antidepressants. While long-term use of antidepressants is recommended when there is a high risk of suicide, psychosis, or recurrent depression [2,4], these situations are most often referred to secondary care with a psychiatrist and rarely occur in primary care [15,16]. A study by our research group showed that in a cohort of 789 primary care patients with depressive symptoms, only 15% of long-term users satisfied the clinical criteria for long-term antidepressant use [13,17].

### First Do No Harm

There is remarkably little research that explores the long-term effects of antidepressant use, although there are indications that it can be harmful, and as of December 2022, 9 antidepressants are listed on the US Food and Drug Administration watch list for potential signals of serious risk [18]. Long-term use is often linked to a range of severe side effects, including increased risks of cardiovascular events [19], gastrointestinal bleeding [20], fractures [21], and diabetes [22]. Psychological dependence is another risk factor, which stems from a user’s perceived need to take antidepressants for fear of a relapse, and that fear, shared by doctors, explains why antidepressant use is often unnecessarily prolonged [23,24], even though it may undermine patients’ autonomy and resilience, resulting in them becoming less likely to self-manage or willing to stop their antidepressant medication.

Crucially, evidence for relapse comes primarily from studies on antidepressant users for whom guidelines recommend continued treatment (ie, those who meet diagnostic criteria for moderate to severe MDD and have been receiving antidepressant treatment for less than 12 months) [25]. In those with milder symptoms, epidemiological research suggests that long-term antidepressant use does not reduce the likelihood of relapse [26] and that inappropriate long-term antidepressant users can safely cease their medication. These findings are supported by a placebo-controlled randomized trial of antidepressant cessation for primary care patients without current depression, which showed a much smaller incidence of relapse than previously thought [27], and are highlighted by a recent meta-analysis on the discontinuation of long-term antidepressant use in patients with MDD or anxiety disorders [28], where patients were actually diagnosed with clinical depression.

Research suggests that antidepressants are more difficult to deprescribe than other medications; however, previous studies have demonstrated limited effectiveness in deprescribing trials of antidepressants compared to other medications [29,30]. Qualitative research, including that from a current National Institute for Health Research–funded study (REDUCE [Reviewing Long-Term Antidepressant Use by Careful Monitoring in Everyday Practice]) [31,32] in the United Kingdom, has identified several key barriers to deprescribing antidepressants, and these include fear of relapse and withdrawal symptoms, beliefs about depression and antidepressants [23,33-35], medical culture [23,33,34,36], and lack of guidelines [24,36,37]. It is, however, essential that the basic therapeutic principle of *do no harm* is respected (ie, do not prescribe or represcribe medication in the absence of a clinical need).

Limiting antidepressant use to only cases in which it is clinically indicated is in line with quality prescribing and will help to reduce costs and associated adverse events (AEs) and improve long-term mental health outcomes for patients [4,38-40]. However, there is a gap in the evidence on the best strategies to address these known barriers to reducing the overprescribing of antidepressants. The difficulties involved in deprescribing antidepressants suggest that a more intensive, patient-focused intervention may help support this process, similar to the psychological support often provided when treatment is initiated.

Shared decision-making focuses on empowering patients to take ownership of their mental health care in order to improve treatment adherence to nonpharmacological interventions known to protect against relapse [41]. Our own preliminary unpublished research has found that patients on long-term antidepressant treatment see a range of potential benefits from antidepressant cessation, such as returning to their natural functioning, improving focus at work, living without medication, and gaining a sense of achievement.

### Enablers of Successful Deprescribing and Development of the Intervention Support Tool

Pilot work [42] has examined the extent to which documented barriers to antidepressant deprescribing exist in the Australian primary care setting. Antidepressant users and GP participants of co-design focus groups were asked to discuss how the identified barriers could be addressed. The following criteria were identified as being essential: a well-defined reason or motivation for ceasing; access to clear, comprehensive information; an easy-to-follow tapering schedule; a good patient-GP relationship; availability of psychological support; and a positive lifestyle in place (Multimedia Appendix 1).

Qualitative studies have also provided valuable insights into the facilitators of antidepressant deprescribing by eliciting patient and provider perspectives, attitudes, and preferences. These studies have highlighted factors, such as patient willingness, feeling ready to stop, confidence to stop, fear of addiction, trust in health care providers, and support networks, as important facilitators of successful deprescribing [23,40,43-45]. However, while qualitative research is instrumental in understanding the contextual nuances surrounding deprescribing, the efficacy of the specific elements of interventions derived from these insights remains largely untested and unevaluated.

Drawing on this evidence, we explored the use of the well-established 5As brief intervention framework (ask, assess, advise, assist, and arrange), which is endorsed by the World Health Organization and the Royal Australian College of General Practitioners for behavior change interventions such as smoking cessation [46,47]. The 5As framework offers a brief, theory-informed framework for identifying need, assessing readiness, providing tailored advice, offering practical help, and arranging follow-up (Multimedia Appendix 2) [48]. As these elements naturally map onto the tasks required to support safe antidepressant tapering, we adapted this approach to develop the WiserAD intervention support tool (IST; a novel, structured approach for deprescribing antidepressants). WiserAD was further informed by the REDUCE study, which took place in UK primary care [31,32]. REDUCE was the first study to include an electronic health intervention with psychological support. It also incorporated a systematic review of interventions to facilitate antidepressant withdrawal and co-production of an internet-supported withdrawal intervention [23,31,49]. Integrated studies have investigated the contexts and mechanisms underlying how the WiserAD IST works [40,50].

### Objectives

The primary objective of the WiserAD trial is to compare the effectiveness of the WiserAD IST (intervention arm) to that of usual care (attention control arm) in helping general practice patients aged 18-75 years, who have been taking antidepressant medication for longer than 12 months and report mild to no symptoms of depression, deprescribe their antidepressant medication within 6 months while maintaining their mental health and well-being.

The secondary objective is to compare the IST to usual care in terms of (1) antidepressant dosage reduction at 3, 6, 12, and 18 months; (2) no worsening of anxiety symptoms at 3, 6, 12, and 18 months; and (3) patient engagement and motivation at 3 and 6 months.

The economic objectives are to determine the cost-effectiveness of deprescribing antidepressant medication using the IST and compare the IST to usual care in terms of the impact on quality of life at 3, 6, 12, and 18 months.

## Methods

### Trial Design

The WiserAD trial is a stratified, single-blind, parallel, 2-arm, superiority randomized controlled trial (RCT) in which primary care patients in Australia are individually allocated to receive either the trial intervention (intervention arm) or usual care (attention control arm).

Participants assigned to the intervention arm receive support (via phone, email, or SMS text messaging) from a registered nurse (WiserAD research nurse) who is trained in mental health treatment, management, and deprescribing. Participants also receive access to the WiserAD IST, which produces a personalized tapering schedule and includes a daily check-in to monitor mood, sleep, and physical activity. Participants are encouraged to discuss their tapering plan with their GPs. Participants assigned to the attention control arm continue with usual care and are free to explore options for deprescribing with their GPs. Both arms receive a link to the *Beyond Blue* website, where they can access information about antidepressant medication [51].

The provision of this particular attention control strengthens the evaluation of the behavioral intervention by providing control participants with access to information that may benefit their involvement in the study, without contaminating the primary or secondary outcomes.

This trial protocol follows the SPIRIT (Standard Protocol Items: Recommendations for Interventional Trials) guidelines [52]. The TIDieR (Template for Intervention Description and Replication) checklist is provided in Checklist 1, and the SPIRIT checklist is provided in Checklist 2.

### Trial Setting

The trial is set within general practices across the state of Victoria, Australia, and includes self-referral through social media advertising across Australia. Participants vary in their demographic, socioeconomic, and geographic characteristics.

### Eligibility and Recruitment

Potential eligible participants are identified using 2 recruitment strategies. The first recruitment strategy identifies potential eligible participants through general practices. General practices are eligible if they (1) are located in Victoria, Australia; (2) have at least 200 patients using antidepressants aged 18‐75 years; (3) are willing to participate in an audit of patient lists to identify patients who meet the criteria for deprescribing; and (4) have capacity to follow the study protocol, including assisting in the deprescribing protocol and risk management of patients.

Potential eligible patients may be systematically identified by GPs either individually during a patient consultation or through an audit of their electronic medical records (EMRs), which identifies patients according to the study eligibility criteria. Two options are available for the GP EMR audit: (1) Torch Recruit and (2) manual search. First, Torch Recruit is a quality improvement system for research that uses an algorithm to identify patients who meet the criteria of interest. The algorithm generates a list of patients with long-term use of antidepressants. This list is audited by GPs to determine which patients currently prescribed antidepressants are potentially eligible to cease their medication through a guided support intervention. The extracted data remain within each specific practice. Second, a manual search of the EMR for potentially eligible patients is conducted by practice staff, and GPs are asked to identify potentially eligible patients.

In the second recruitment strategy, participants may self-refer to the trial by completing an online expression-of-interest form advertised on social media sites (Facebook and Instagram). The form collects only essential contact details, including GP name and contact details. Individuals who complete the form are contacted by a member of the research team via a phone call to explain the study in full and proceed with eligibility screening. Individuals are asked to provide their GPs’ contact details so that the GPs are informed of their patients’ participation, maintain clinical support, and are provided with information about the trial.

Eligibility criteria for individuals are as follows: (1) long-term use of antidepressants (defined as continuous prescription for ≥12 months) and current active prescription for selective serotonin reuptake inhibitors or serotonin and norepinephrine reuptake inhibitors for the treatment of depression and anxiety; (2) age of 18‐75 years; (3) stable on antidepressants for ≥12 months and no depressive episodes, as documented by self-report and a GP; (4) no use of antidepressants (or antipsychotics) for diagnoses other than depression or anxiety; (5) no history of psychosis, bipolar disorder, or obsessive compulsive disorder; (6) proficiency in English to provide informed consent; (7) agreement to consider reviewing antidepressant use; and (8) no or mild depressive symptoms (defined as having a total Patient Health Questionnaire-9 [PHQ-9] score of <10 for depressive symptoms) [53].

Patients identified as potentially eligible for the trial at the GP sites are invited to participate through an email, SMS text message, or letter sent by their regular GP. GPs are invited to refer patients at the point of care during a routine visit if they meet the eligibility criteria. Through this correspondence, patients are asked to contact the research team directly.

All patients who express an interest in participating are invited to meet with a member of the research team in-person, through online conference software, or by telephone to discuss the study in more detail and determine if they meet the eligibility criteria.

A key part of the recruitment process is the willingness of potential participants to review and consider deprescribing their antidepressant medication. Participants are only enrolled if they understand that they will be required to review their medications. Participants’ feelings about and relationships with their medications are also explored within the intervention measures recorded after randomization.

The full eligibility screening process is carried out in 2 stages to ensure that participants’ mood remains stable over 2 time points (2 weeks apart). The first screening is conducted through the WiserAD website, where a researcher creates a user account and completes the first screening questionnaire with the participant. The second screening occurs 2 weeks later and may also be conducted with a researcher; alternatively, participants may log in and complete the screening tool independently, if preferred.

The second eligibility screening confirms the following: (1) no or mild depressive symptoms (defined as having a total PHQ-9 score of <10 for depressive symptoms) [53]; (2) low risk of suicide or self-harm (PHQ-9) [53]; (3) agreement to be randomized in the study; and (4) willingness to provide informed consent.

For individuals who do not meet the eligibility criteria, no personal data are retained, except for demographics used to characterize the sample.

### Exclusion Criteria

Patients who are currently experiencing or expecting to experience a major life event in the next 3 months (eg, trauma, grief, unemployment, financial stress, and major health issues) or who do not have daily access to the internet are not eligible to take part in the trial.

### Consent

In line with International Council on Harmonisation of Technical Requirements for Registration of Pharmaceuticals for Human Use–Good Clinical Practice (ICH-GCP) [54] regulations, patients who are eligible and willing to participate are provided with a patient information sheet (PIS) that informs patients about the study purpose, their involvement in the study, data management, and their right to withdraw. The PIS also informs patients that they need to provide additional consent to allow access to their routinely collected health service use data and prescription data for the study’s health economic analysis. It is made clear that they can decline this consent without it affecting their participation in the study or the services they usually receive from their GPs. Once participants are determined to be eligible and have provided verbal agreement to take part in the trial, they can electronically consent to the study via their user account. A digital record of the consent is retained by the research team.

Participants are sent a separate PIS and consent form to allow access to their medical service use data and prescription medication data (Medicare Benefits Schedule [MBS] and Pharmaceutical Benefits Scheme [PBS] data) collected by the Australian Government. Participants are encouraged to read this information and, if they agree to participate, complete the consent form and return it to the research team using a postage-paid envelope. Participants can decline consent to access their collected health service use data and prescription data without it affecting their participation in the study.

After providing consent, all participants are asked to complete a set of baseline standardized measures to assess current mood, anxiety, beliefs about their medication, satisfaction with their current treatment and self-management, and quality of life (Table 1). These data are collected within a Research Electronic Data Capture (REDCap) [55] database embedded within the WiserAD website and linked through a unique identifier via an application programming interface. Participants who are ineligible due to low mood but keen to take part in the study will be advised to reapply for participation in 8‐12 weeks, depending on the severity of their symptoms at the time of eligibility screening.

**Table 1.** Measures in the WiserAD trial.

Measure	Purpose	Time of assessment				
		Baseline	3 months	6 months	12 months	18 months
Demographics	Gender at birth, year of birth, and postcode	✓	—^a^	—	—	—
PHQ-9^b^ [53]	Depressive symptoms	✓	✓	✓	✓	✓
GAD-7^c^ [56]	Anxiety symptoms	✓	✓	✓	✓	✓
BMQ^d^ [57]	Beliefs about medications	✓	✓	✓	✓	✓
PAM^e^ [58]	Patient activation	✓	✓	✓	✓	✓
AQoL-4D^f^ [59]	Quality of life	✓	✓	✓	✓	✓
RUQ4^g^ [60]	Health care use	✓	✓	✓	✓	✓
Signs and symptoms	Common side effects	✓	✓	✓	✓	✓
Medications	Current medications	✓	✓	✓	✓	✓
MBS^h^	Medical benefits	—	—	—	—	✓
PBS^i^	Pharmaceutical benefits	—	—	—	—	✓
AMT^j^ [61]	Accountability	✓	✓	✓	—	—
UES-SF^k^ [62]	User engagement	✓	✓	✓	✓	✓

a Not applicable.

b PHQ-9: Patient Health Questionnaire-9.

c GAD-7: Generalized Anxiety Disorder-7.

d BMQ: Beliefs About Medicines.

e PAM: Patient Activation Measure.

f AQoL-4D: Assessment of Quality of Life Scale.

g RUQ4: Resource Use Questionnaire-4.

h MBS: Medicare Benefits Schedule.

i PBS: Pharmaceutical Benefits Scheme.

j AMT: Accountability Measurement Tool.

k UES-SF: User Engagement Scale-Short Form.

### Randomization and Allocation Concealment

Once eligibility is confirmed, consent is provided, and baseline measures are completed, participant details are entered into a randomization system embedded within the WiserAD website. Allocation is triggered when the participant confirms completion of the REDCap baseline assessment by clicking a button on the WiserAD website. Participants are assigned with equal probability to the 2 trial arms using a computer-generated allocation sequence, with stratification by general practice for participants identified via general practices (recruitment strategy 1) or by state of residence for self-referring participants (recruitment strategy 2). An allocation sequence is generated using random permuted block sizes within each stratum. To ensure concealment, the block sizes are not disclosed until after trial recruitment is complete. A trial statistician who is not involved in participant recruitment or data collection generates a random allocation schedule. Research staff who are involved in recruitment and intervention delivery may be aware of participant allocation when necessary for trial conduct. Data analysts and other research team members who do not require access to allocation information remain blinded until completion of the analysis. GPs are not informed about participants’ treatment allocations; however, complete blinding of GPs is not possible as they may make inferences about allocation. This is not expected to impact trial conduct, outcomes, or analysis integrity. The trial manager does not communicate with the research team about participant allocation during the trial, and any cases of unblinding are recorded.

The random allocation sequence is embedded within the website, ensuring allocation concealment. Only the trial manager or their nominee has password access to the random allocation of participants. Participants are not blinded to their trial arm assignment. Trial arm allocation remains concealed from the research team until recruitment and follow-up are complete and the cleaned dataset is locked for analysis.

### Randomization Codes

Investigators may obtain the allocation information of participants through password-protected access after the trial has concluded and the primary outcome analysis is complete. In the case of a medical emergency or serious AE, the trial manager will be asked to identify the trial arm. Following data collection, an analysis will be performed to determine if incidences of unblinding are equal in the 2 trial arms.

### Interventions

#### Attention Control Arm

Participants allocated to the attention control arm receive usual care with their GPs (ie, continued access to routine GP consultations, prescription renewals, and standard clinical management) and access to an online antidepressant fact sheet within the Beyond Blue website [51], an Australian information and support website for better mental health. This fact sheet provides educational material relevant to enrollment in the study, but participants do not receive advice regarding stopping or continuing their medication.

#### Intervention (Active) Arm

The WiserAD website has been designed through the collaboration of mental health, primary care, implementation, service user, and digital design expertise to ensure that patients who are randomized to this arm are suitably informed, eligible, supported, and prepared for antidepressant deprescribing with minimal risk.

Within 3 business days after being randomly assigned to the intervention arm, a WiserAD research nurse calls participants and guides them through the support tool. The support tool provides assessment and advice tools to develop a personalized support strategy during tapering (Multimedia Appendix 3). Participants then complete their user account setup by entering the metrics they use to monitor their own mental health, such as sleep quality, current mood, and activities. Potential reasons for fluctuations in these metrics can also be logged (Multimedia Appendix 3) and flagged by participants when these indicate a drop in mood relative to that identified at the start of the intervention. Participants complete the tapering schedule and user account independently and/or with the WiserAD research nurse. The WiserAD research nurse follows up with participants at routine and mutually agreed times (eg, 1-week, 2-week, or monthly intervals) for a period of no more than 6 months.

GPs are informed about patients allocated to the intervention arm of the study via an email from the research team. Participants are encouraged to discuss any planned medication changes with their GPs, seek clinical advice if they have concerns, and receive corresponding prescriptions according to the tapering plan. Participants may continue in the study without explicit permission from their GPs. Their GPs can modify the tapering schedule if they feel that it is not appropriate and/or if the WiserAD research nurse informs them that their patients are not responding well to the reduced dosage, as indicated by the daily tracker. A practitioner-facing interface in the WiserAD website allows the study team and the GPs to oversee the progress of patients.

The daily symptom tracker is monitored by the WiserAD research nurse or trial manager to closely monitor the mood and response of patients to the tapering schedule.

A standard operating procedure (SOP) is followed for patients who do not complete the daily tracker, show a major decline in current mood, or show symptoms that have been identified as warning signs for a declining mood state in their daily tracker (see the Risk Management section). As part of the SOP, the PHQ-9 [53] is completed to assess the severity of depressive symptoms.

Participants continue to have access to the WiserAD website until trial completion (after all trial participants complete the final follow-up survey). They also receive a link to access the antidepressant factsheet within the Beyond Blue website.

### Outcomes

#### Overview

Outcome measures are assessed at 3, 6, 12, and 18 months after randomization to allow for shorter- and longer-term monitoring and to inform implementation and prescribing guidelines (Table 1). All measures are collected via a REDCap link sent to participants; however, they may also complete these assessments over the phone with a researcher.

#### Primary Outcome

The primary outcome is the difference in the proportion of eligible individuals successfully ceasing antidepressant use at 6 months between the intervention and attention control arms. Successful cessation is defined as no antidepressant use and the absence of clinically significant depressive symptoms. Antidepressant use is assessed via self-report (ceased/not ceased) and validated where possible using PBS data. Depressive symptom severity is assessed using the PHQ-9, and the absence of clinically significant symptoms is defined as a PHQ-9 score of <10. The PHQ-9 provides a conservative estimate of depressive symptom severity, and thus, patients are less likely to be misclassified as having an absence of clinically significant depressive symptoms when they report depressive symptoms with a PHQ-9 score of >10, which indicates moderate/severe depressive symptoms. The PHQ-9 is commonly used in general practice trials.

#### Secondary Outcomes

Secondary outcomes are assessed to capture any changes in antidepressant use and longer-term management, mood, and activities. These include differences between the intervention and attention control arms in (1) the proportion of patients maintaining a reduced antidepressant dosage at 6 months after randomization; (2) the proportion of patients successfully ceasing antidepressant use at 3, 12, and 18 months; (3) mean anxiety symptoms at 3, 6, 12, and 18 months measured using the Generalized Anxiety Disorder-7 (GAD-7) [56]; and (4) mean patient engagement and motivation at 3 and 6 months measured using the Patient Activation Measure (PAM) [58,63].

### Economic Evaluation

A comprehensive economic evaluation of quality of life and health care use (medical and pharmaceutical) will be conducted using a study-specific self-report Resource Use Questionnaire-4 (RUQ4) adapted from a questionnaire used in a previous trial conducted by our group [64,65]. The RUQ4 will record the type, frequency, and duration of health service use by participants through direct and indirect cost savings in the preceding 3 months and will be collected at 3, 6, 12, and 18 months. Through the assessment of measures of social activities, such as work and benefits, the impact of mental health can be assessed. The RUQ4 will be integrated into the website and will follow a conditional logic model, where subsequent questions will only be presented if a participant’s previous response requires further information. This approach is intended to help with the completion of the questionnaire by reducing the burden on participants.

The Assessment of Quality of Life-4D (AQoL-4D), a validated measure, will be used to assess the impact of mental and physical health on everyday quality of life [59]. For participants who provide additional consent, reliable health service use data will be obtained from the MBS and PBS. These records provide supporting data and information regarding primary and secondary care visits and prescription medications through Australia’s universal health care system.

The actual cost of the intervention will be determined and captured using financial and provider records.

Mediators will be measured at baseline and 3, 6, 12, and 18 months. To understand possible mediators, the following will be recorded: (1) participant beliefs and motivations related to their relationships with medications, which will be measured using the Beliefs about Medicines Questionnaire (BMQ) [57]; (2) accountability and engagement, which will be measured using the Accountability Measurement Tool (AMT) [61] and User Engagement Scale-Short Form (UES-SF) [62], respectively; and (3) signs and symptoms typically associated with reducing antidepressants, which will be assessed (text responses) during contact with the WiserAD research nurse.

Other measures include data from the daily tracker, retention, and compliance with the tapering schedule (assessed using analytic data collected on the WiserAD website).

### Process Evaluation (Realist Approach)

The intervention has been evaluated with a realist approach [66,67] to explore the experiences of 13 participants, using qualitative interviews (conducted over the phone or via Zoom videoconference software) and quantitative data from the baseline and 3-month follow-up surveys [40]. The interviews followed a narrative approach to elicit rich responses with minimal prompting [68] and focused on participant engagement with the study and study website and the expectations for online tapering support. The evaluation followed the Pawson and Tilley realist approach [66] and RAMESES II (Realist and Meta-Narrative Evidence Syntheses: Evolving Standards) guidance, where the analysis used an abductive realist logic to generate and refine context-mechanism-outcome configurations, with mechanisms conceptualized as interactions between program resources and participant reasoning [69]. Full methodological details, including the interview guide and analytic procedure, are provided in a published realist-evaluation protocol and related outcomes paper [40,50].

### Sample Size

We require 340 patients at baseline (170 patients per arm) for 80% power with a 5% significance level (2-sided test) to detect a 20% difference in the proportion of patients who successfully cease antidepressant use at 6 months between the intervention and attention control arms (36% vs 16%), after allowing for up to 50% attrition at 6 months (primary outcome). The sample size is based on the proportion of participants who cease antidepressant use at 6 months and for whom the PHQ-9 score is <10 (primary outcome).

The initial target sample size was 312 patients at baseline (156 patients per arm) for 90% power (5% significance level and allowing 30% attrition over 18 months). As of June 25, 2025, the 6-month survey response rate was closer to 50%, which was lower than anticipated. Thus, with approval from the Data Monitoring Committee’s independent statistician, the final target sample size was increased from 312 to 340 while maintaining at least 80% power for the primary outcome.

In a 10-year longitudinal study of general practice attendees, the estimated natural rate of cessation among patients with PHQ-9 scores below the threshold was between 10% and 16% annually [64,65,70]. Given that willingness to review medication is an inclusion criterion for this study, we expect the cessation rate in the attention control arm to be at the high end of this range (16%). Published primary care deprescribing interventions show no improvement over this natural cessation [71]; thus, we have determined our minimally important effect size of 20% based on studies of more intensive (albeit nonprimary care) deprescribing interventions, which have achieved 36% antidepressant cessation [72]. To achieve the target sample size, we need to invite 940 eligible patients. This value is based on a previous deprescribing RCT [27], which demonstrated that approximately one-third of eligible patients consented to a deprescribing trial.

### Data Collection and Management

All data of eligible trial participants will be collected through the study website, WiserAD [73], and the embedded University of Melbourne REDCap system [55], including consent forms and health economics questionnaires.

The WiserAD website is a secure, password-protected online platform that uses encrypted HTTPS connections to ensure data privacy and integrity. REDCap is a secure, web-based platform designed to support data capture for research studies, providing (1) an intuitive interface for validated data entry; (2) audit trails to track data manipulation and export procedures; (3) automated export functions to common statistical packages; and (4) tools for integration and interoperability with external data sources.

Trained research assistants will enter data using individual, password-protected accounts, and participants will receive a unique login and password to access the study website and IST, followed by secure, personalized links to complete follow-up study measures in REDCap. Each participant will be assigned a unique ID to link all data throughout the study while protecting confidentiality. Participants will receive follow-up reminders at scheduled time points, as described in the Retention section.

All study raw data will be downloaded from secure Australian-based servers and stored on password-protected servers at the University of Melbourne, with access restricted to authorized study team members. Data transfers will occur via encrypted HTTPS connections, and daily automated backups will be securely stored. Data exported from WiserAD and REDCap for analysis may initially contain identifying information, such as contact details; however, these identifiers will be removed during data cleaning to produce deidentified datasets for all analyses.

Data cleaning and processing will be performed using Stata (version 17). Datasets from different collection points will be merged to form the final analysis dataset. Data will be scored and processed according to the respective questionnaire manuals and scoring guidelines to ensure accuracy, consistency, and validity. Data quality will be maintained through built-in validation rules and periodic quality assurance checks by the data manager, with all access events and changes automatically logged.

Deidentified datasets will be retained for a minimum of 5 years following publication of study results, after which they will be securely archived or destroyed in accordance with the University’s research data management policy.

### Retention

The following steps are taken to limit missing responses for the primary outcome and minimize participant attrition:

1. Reminder emails or text messages are sent to all participants 72 and 24 hours prior to their follow-up due date. If they do not complete the follow-up, another text or email is sent once a week for 2 weeks. If they still fail to respond, a researcher calls them via telephone up to a maximum of 4 times over the next 2 weeks. Participants also have the option to complete the follow-up measures over the phone with a researcher. Participants are declared nonresponders for the follow-up time point if they do not respond within 1 month of the time point. Only participants who actively inform us that they wish to withdraw are marked as withdrawn in the study log; otherwise, they are assumed to be lost to follow-up at each time point.
2. Participants in the intervention arm who are not responding to their daily symptom trackers are contacted according to the SOP for risk management. If it becomes clear that they are well but not willing to complete the tracker, the WiserAD research nurse discusses how to proceed, initially suggesting to complete the tracker every other day.
3. Participants who do not consent to the collection of their health economics information are reminded of this option at each follow-up time point.
4. Participants who complete their routine follow-up questionnaires (at least the primary measure) are eligible to participate in a monthly prize draw to win a shopping voucher worth AUD $100 (approximately US $71).

People who wish to take part in the study but are not eligible may be asked to consent to take part in short, anonymous surveys about their current use of antidepressants and their understanding of antidepressants, and may be offered a chance to discuss these with a researcher in a series of qualitative interviews designed to gauge participants’ thoughts and feelings about their medications and why they consider stopping. These participants are not included in the main trial analysis but may provide valuable qualitative evidence for the deprescribing literature.

### Statistical Methods for the Primary and Secondary Objectives

Descriptive statistics will be used to review the demographic characteristics of the participants and assess the balance of participants within the 2 trial arms. We will use generalized linear models with identity and logit link functions and binomial distributions for both models to estimate the between-arm difference in the proportion of participants ceasing antidepressant use and having a PHQ score of <10 (absolute measure) and to assess the odds ratio (relative measure) at each time point (3, 6, 12, and 18 months), respectively. Both models will use generalized estimating equations with robust SEs (accounting for repeated outcome measures on individuals) and include stratification factors (general practice/state) and time as covariates. Multiple imputation may be used to handle missing data, and this is contingent upon an examination of the patterns of the missing data in a blinded review of the data. A similar approach will be undertaken for secondary binary outcomes. For secondary continuous outcomes, a constrained longitudinal data analysis will be used, with response variables consisting of all outcomes measured at each time point (0, 3, 6, 12, and 18 months). Linear mixed-effects models using restricted maximum likelihood, with random intercepts for individuals, will be used to estimate the difference in means between the trial arms at each measurement time point. Regression analyses will adjust for baseline outcome measures (when appropriate), the stratification variable (site/state), time, and the trial arm (intervention/attention control). A 2-way interaction between arm and time will be included, with the exception of the baseline, where means are constrained to equality. The estimated intervention effect will be reported as between-arm differences in proportions and odds ratios for binary outcomes and between-arm differences in means for continuous outcomes at each time point (3, 6, 12, and 18 months), with their respective 95% CIs and *P* values. There will be no adjustments for handling the multiplicity of testing, and no formal interim analysis is planned.

Primary analyses will use an intention-to-treat strategy, where all individuals will be analyzed in the trial arm to which they have been randomly allocated [74]. Additional sensitivity analyses will include adjustment of prespecified prognostic baseline variables (eg, age and duration of antidepressant use) in the regression models, using complete cases only and using pattern-mixture models, to assess the robustness of the missing data assumption. A detailed statistical analysis plan will be developed to outline supplementary analyses, such as methods for handling missing data and assessing the impact of nonadherence on the estimated intervention effect, and subgroup and exploratory analyses. The statistical analysis plan will be made available prior to the commencement of any statistical analysis. Analyses will be conducted in Stata 17.0 or later.

### Economic Analysis

A cost-consequences analysis will compare the incremental costs of the intervention to the full spectrum of outcomes included in the study. A series of cost-effectiveness ratios will be determined rather than just one—an approach that is useful to decision-makers. The inclusion of a quality-of-life measure (AQoL-4D) enables a cost-utility analysis to be undertaken, where outcomes are expressed in generic quality-adjusted life years (QALYs), thereby allowing practical judgments to be made regarding the value-for-money credentials of the intervention. For example, in Australia, cost-effectiveness ratios falling below AUD $50,000/QALY (approximately US $35,750/QALY) are deemed good value for money [65].

The evaluation will first assess the value of any changes to the use of health care resources over the study period for both treatment arms (including costs of delivering the intervention) and then compare any antidepressant or additional costs to the outcomes achieved. While we expect medication use to decrease, we will assess the value of the use of all services (using publicly available unit costs where possible). The economic analysis will be conducted from a societal perspective, including all costs (even those outside the health sector) and productivity impacts. Secondary analyses from narrower perspectives (eg, health) will also be undertaken to address the interests of different stakeholders.

We will use standardized economic evaluation techniques, including incremental analyses of mean differences and bootstrapping, to determine CIs. Regression techniques using a net-monetary benefit framework will also be used to investigate subgroup effects and the potential predictors of differences in costs and benefits, as well as overall cost-effectiveness. If the intervention is found to be effective, we will determine the lifetime and population cost-effectiveness of the intervention using modeling techniques refined by health economists from the study team.

### Monitoring

In addition to monthly working committee meetings, the trial will be monitored quarterly by a trial steering committee. This committee comprises the chief and associate investigators, including a biostatistician and a health economist. A research fellow and the trial manager will also attend meetings. An independent data monitoring committee with an established charter will meet regularly to track progress and recruitment, review AEs, and monitor the protocol design. The data monitoring committee is comprised of experts in the fields of clinical trials, primary care, and biostatistics, and it will provide feedback to the trial steering committee.

### Risk Management

The clinical care of all participants will be managed by their treating GPs throughout the trial.

There is no planned assessment of safety within the study design, and unless participants report any intercurrent illnesses or AEs directly to the study team, it is unlikely that these will be systematically recorded. However, if a participant scores 2 or 3 on the PHQ-9 item “thoughts that you would be better off dead or of hurting yourself in some way,” the researcher will flag the finding or an online recommendation will be provided to consult their GP to discuss the finding. The research team will also be notified of this response and will attempt to contact the participant. Moreover, the trial manager or WiserAD research nurse will break the identity code and contact the GP directly to inform them about the finding and recommend that they contact the participant.

Participants experiencing a higher number of symptoms than previously indicated or showing signs of worsening mood will be contacted by the WiserAD research nurse for a structured risk assessment. Follow-up protocols, which include alerting the GP, will be triggered for participants identified to be at high risk of self-harm. A consultant psychiatrist from the chief investigator team will be available to provide psychiatric input upon request by the study team or participating GPs. Regardless of their allocation, all participants will be provided with the number for Lifeline and will be encouraged to discuss any concerns with their GPs. Finally, for participants in the attention control arm, involvement in the study is not expected to pose risks beyond those of usual GP care, as these participants will not be advised to discontinue their antidepressant medication and will continue to receive treatment as usual.

### AE Reporting

AEs and serious AEs will be recorded and reported in line with ICH-GCP guidelines and the University of Melbourne guidelines. The report will include the severity of the event, relatedness to the study intervention, anticipation of the event, and action taken, and it will be sent for review by the steering committee. Serious AEs will be reported in a timely manner to the University of Melbourne Human Research Ethics Committee (HREC) and evaluated by the study’s clinical lead and the data monitoring committee.

### Ethical Considerations

This protocol has been approved by The University of Melbourne HREC (ID: 20558). The Commonwealth Department of Human Services has approved the collection of routine health service use data within the MBS and PBS for those participants who have provided additional consent. Informed consent will be obtained from all participants (see the Consent section). A well-established distress protocol, in use by the research team for over a decade, will be in place to manage any potential participant distress during the study. This protocol outlines clear procedures for researchers to follow if a participant experiences emotional distress, ensuring that prompt and appropriate support is provided. Participants will not receive direct financial compensation for taking part in the study. However, any participant who completes a follow-up survey will be automatically entered into a monthly random draw to win a gift card worth AUD $100 (approximately US $71). Data will be assigned a unique identifier, and any directly identifying information will be stored separately on secure, password-protected servers with access restricted to authorized study personnel. Study results will be reported only in aggregate form. Any substantial amendments to the protocol will be sent for approval by the University of Melbourne HREC before implementation. Trial results will be disseminated in peer-reviewed publications, conference presentations, reports in trial registries, and a plain language newsletter for participants.

### Trial Registration

The study has been prospectively registered with the Australian & New Zealand Clinical Trials Registry (ACTRN 12622000567729), the International Registry for Clinical Trials (ISRCTN 11562922), and ClinicalTrials.gov (NCT05355025).

## Results

Participant recruitment for the WiserAD trial commenced in May 2022 and and was completed in January 2026. Trial outcomes will be reported in peer-reviewed journals in Febraury 2027, according to the CONSORT guidelines for RCTs [75]. Participants will be asked if they wish to receive the trial results upon completion, and those who consent will receive updates through a plain language newsletter.

## Discussion

### Anticipated Findings

The WiserAD IST aims to increase the proportion of participants who successfully discontinue long-term antidepressant use while maintaining mental health at 6 months compared with usual care. The trial also examines whether anxiety symptoms are maintained, patient activation increases, and beliefs about medication shift toward a neutral stance. The trial describes patterns of antidepressant use and longer-term management. If the WiserAD IST maintains patient mental health while improving appropriate discontinuation or dose reduction, it will offer a practicable, patient-centered pathway for deprescribing that balances safety with scalability. If outcomes are modest, quantitative and qualitative findings will indicate which components require strengthening to achieve larger, sustained reductions in inappropriate long-term antidepressant use.

### Comparison With Prior Work

To date, there has been limited research into the process of antidepressant deprescribing and the optimal support mechanisms for patients and health care providers to safely and effectively complete this process [28]. The completed PANDA (Prescribing Antidepressants Appropriately) RCT in the Netherlands was the first published trial of an intervention aimed at ceasing antidepressant use [30,76]. The PANDA study highlighted the challenges inherent in antidepressant deprescribing for both patients and health care providers. GPs of 70 antidepressant users from 45 general practices received an advisory letter, a tapering plan, and an information sheet with current guidelines on antidepressant tapering and withdrawal from the research team. Only 4 (6%) intervention participants successfully discontinued antidepressant use compared with 6 (8%) control participants. Additionally, 56% of participants in the intervention group rejected the recommendation to discontinue after shared decision-making with their GPs. Participants cited several reasons for not adhering to the deprescribing recommendations, including fear of relapse, negative previous experiences with tapering, presence of depressive symptoms, and a desire for a second opinion [77].

The intervention in the ANTLER (Antidepressants to Prevent Relapse in Depression) trial is an important comparator to our intervention as it involved a blinded taper-to-placebo design, in which active medication and matched placebo were encapsulated to ensure that all capsules were visually indistinguishable [71]. The findings showed a substantially higher relapse rate in the discontinuation group (56%) than in the maintenance group (39%). Regarding secondary outcomes, there were increases in mean depressive and anxiety symptom scores and withdrawal symptoms, particularly around the early weeks after tapering, and adherence was lower in the discontinuation arm.

The REDUCE study in the United Kingdom represents the first electronic health intervention with psychological support tailored to aid antidepressant deprescribing in primary care [32]. The REDUCE study demonstrated noninferiority regarding depressive symptoms and modest improvements in withdrawal symptoms and well-being; however, there was no statistically significant increase in the discontinuation rate compared with active review alone. In Australia, the RELEASE (Redressing Long-Term Antidepressant Use in General Practice) trial is currently ongoing and focuses on hyperbolic tapering and GP education [78].

The WiserAD trial is positioned to address the limitations identified in the current literature and to build on emerging evidence about how best to support patients and clinicians. Trials to date have highlighted that (1) simple recommendations alone rarely change entrenched prescribing (PANDA) [76], (2) stopping maintenance antidepressants carries a measurable risk of relapse for some patients (ANTLER) [71], and (3) digitally delivered or telephone-supported interventions can improve tolerability and preserve well-being but may not lead to sustained increases in cessation (REDUCE) [32]. The RELEASE trial further illustrates the move toward multistrategy, implementation-focused trials that combine patient resources, hyperbolic tapering protocols, and practice-level supports [78].

The WiserAD tool differs from these approaches in several ways. First, it is a co-designed, patient-facing digital IST that integrates tailored educational materials, decision aids, and a personalized tapering algorithm informed by contemporary hyperbolic tapering guidance, together with structured nurse support. Second, WiserAD places emphasis on dosing personalization and relational-clinical support delivered via a scalable web platform rather than relying on practitioner mailouts or practice-level audits alone. Third, WiserAD translates qualitative insights into an intervention design where the intervention distinguishes withdrawal from relapse (via nurse support), offers decision aids to support shared decision-making, uses hyperbolic tapering in a personalized algorithm, and provides nurse support to address patient concerns in real time. Fourth, the embedded realist evaluation is unique in exploring how, for whom, and in what circumstances the WiserAD IST operates to maintain wellness and support deprescribing [40].

### Strengths and Limitations

A primary strength of the WiserAD approach for antidepressant deprescribing is its patient-facing design, which engages patients in their own health care and increases the potential for behavior change. The personalization of the tapering schedule with nurse support is novel and has implications for the implementation of deprescribing in clinical practice. The intervention was also co-designed with patients and GPs. The patient-facing nature of the IST improves user acceptability and increases the likelihood that the content and delivery mode will be meaningful and scalable in real-world settings.

Participants who enroll in the trial may already have an interest in deprescribing their antidepressants, and some participants allocated to the attention control arm may attempt to taper their medications. However, this would allow for a deeper understanding of the differences between the WiserAD IST and antidepressant deprescribing in a naturalistic and usual care setting.

### Conclusions

The WiserAD IST is a novel, co-designed, patient-facing digital tool that combines personalized tapering, educational materials, decision aids, and structured nurse support. This novel IST is scalable to routine primary care practice while having minimal impact on GP workload and providing deprescribing guidelines and practical support. The WiserAD IST facilitates safe and appropriate dose reduction or discontinuation without clinically meaningful worsening of depressive symptoms. Alongside the outcome data, the embedded realist evaluation identifies the mechanisms for implementation of the IST in practice. This research holds high potential to inform Australian mental health policy and clinical practice.

## Supplementary material

10.2196/81858 Multimedia Appendix 1 Co-design focus groups.

10.2196/81858 Multimedia Appendix 2 The WiserAD 5As approach.

10.2196/81858 Multimedia Appendix 3 WiserAD screenshots.

10.2196/81858 Checklist 1 TIDieR checklist

10.2196/81858 Checklist 2 SPIRIT 2025 checklist.

## Abbreviations

AE: adverse event
AMT: Accountability Measurement Tool
ANTLER: Antidepressants to Prevent Relapse in Depression
AQoL-4D: Assessment of Quality of Life-4D
BMQ: Beliefs about Medicines Questionnaire
EMR: electronic medical record
GAD-7: Generalized Anxiety Disorder-7
GP: general practitioner
HREC: Human Research Ethics Committee
ICH-GCP: International Council on Harmonization of Technical Requirements for Registration of Pharmaceuticals for Human Use–Good Clinical Practice
IST: intervention support tool
MBS: Medicare Benefits Schedule
MDD: major depressive disorder
PAM: Patient Activation Measure
PANDA: Prescribing Antidepressants Appropriately
PBS: Pharmaceutical Benefits Scheme
PHQ-9: Patient Health Questionnaire-9
PIS: patient information sheet
QALYs: quality-adjusted life years
RAMESES: Realist and Meta-Narrative Evidence Syntheses: Evolving Standards
RCT: randomized controlled trial
REDCap: Research Electronic Data Capture
REDUCE: Reviewing Long-Term Antidepressant Use by Careful Monitoring in Everyday Practice
RUQ4: Resource Use Questionnaire-4
SOP: standard operating procedure
SPIRIT: Standard Protocol Items: Recommendations for Interventional Trials
TIDieR: Template for Intervention Description and Replication
UES-SF: User Engagement Scale-Short Form
