# Supplementary material for: Structured Online Support to Inform and Assist Antidepressant Deprescribing in Primary Care (WiserAD Tool): Protocol for a Pragmatic Randomized Controlled Trial
Source: JMIR Res Protoc. 2026 Aug 7;15:e81858. doi: 10.2196/81858 (PMC13449978; doi:10.2196/81858)
Supplement: Supplementary file 1 [file resprot-v15-e81858-s001.docx]

## Multimedia Appendix 1: Overview of the co-design focus groups that informed the development of the WiserAD IST.

In 2016, two separate focus groups were conducted with consenting Victorian (Australia) GPs (*n* = 9) and individuals (patients) with a history of long-term antidepressant use (current or past) (*n* = 9). The majority of patient participants identified as female (*n* = 6, 67%) and were aged between 18 and 35 years of age (*n* = 5, 56%). All patient participants had a tertiary education. Most GP participants identified as male (*n* = 6, 67%), were aged 50 years and older (*n* = 8, 89%) and had been practicing as a GP for an average of 27 years. The focus groups were facilitated by two academic GPs (Prof Jane Gunn and Dr Christopher Otjes) and a research psychologist (Dr Susie Fletcher).

The study aimed to:

1. Identify how, in the absence of detailed guidelines, GPs currently manage antidepressant cessation;
2. Understand the barriers and facilitators to antidepressant cessation from GP and patient perspectives;
3. Develop a conceptual framework to guide design specifications for prototype development from the findings of the study.

Broad topic guides were used to facilitate discussion on long-term antidepressant use, perceived issues with ceasing antidepressants, and the desired components of an intervention to assist in the cessation process. Question prompts used in the focus groups can be found in Table S1.

**Table S1** Focus Group Questions for Patients and GPs About Antidepressant Treatment

*Focus Group Questions for Patients and GPs About Antidepressant Treatment*

| Patient focus groups | GP focus groups |
| --- | --- |
| What comes to mind when you think of antidepressants? | What comes to mind when you think of long-term antidepressant use? |
| Do you feel like you know enough about ADs? | What factors are important when prescribing antidepressants? |
| What comes to mind when you think of stopping antidepressants? | What challenges are there in prescribing antidepressants? |
| What would be the reason to stop/not stop? | How long should people take antidepressants? |
| Where would you go for information? | What factors make you consider recommending ceasing antidepressants? |
| What would be some challenges to stopping? | What challenges/barriers do you encounter when assisting patients to cease antidepressants? |
| What would help to stop? |  |

The focus groups were audio-recorded, transcribed, and reviewed by the research team both independently and as a group to identify key themes. While there were some similarities between patients and GPs, important differences in their views about antidepressant cessation also emerged. Patients desired more information about antidepressants and their cessation than GPs thought was needed, and a more structured cessation program. Patients also desired more support than would be feasible for most GPs to provide in usual practice. Elements for a deprescribing intervention appeared to be: 1. Requiring information, 2. Alternative management strategies, 3. Clear tapering schedule, and 4. Proactive support and monitoring. The study was funded by a Therapeutic Guidelines Ltd (TGL)/RACGP Research Grant (2016).
