# Supplementary material for: Structured Online Support to Inform and Assist Antidepressant Deprescribing in Primary Care (WiserAD Tool): Protocol for a Pragmatic Randomized Controlled Trial
Source: JMIR Res Protoc. 2026 Aug 7;15:e81858. doi: 10.2196/81858 (PMC13449978; doi:10.2196/81858)
Supplement: Supplementary file 2 [file resprot-v15-e81858-s002.docx]

Multimedia Appendix 2: The WiserAD 5As approach (adapted from the US Public Health Service for tobacco use)

- *Assessment survey:* This component asks about current and past depressive symptoms, antidepressant name and dose, views on antidepressant use, availability of formal and informal support, and general health (e.g. nutrition, exercise, sleep, and stress management). Before attempting cessation, patients receive feedback about where things are going well and where improvements could be antidepressant.
- *Antidepressant Advice:* In this component, patients learn more about their specific antidepressant (long-term use, risks, benefits) and the process of de-prescribing (safety, rationale behind tapering, discontinuation symptoms).
- *Assist:* Three sub-components help patients create a personalized plan to cease antidepressants:

*Prepare:* Based on their own data, patients are guided to select management strategies tailored to their needs and emotional wellbeing (e.g. exercise; social and psychological support) and include links to relevant websites, self-help apps, and services. Patients are given general strategies for managing withdrawal symptoms and are encouraged to discuss their plan with their GP.

*Schedule:* Patients select a date to start their antidepressant taper. While the date is flexible, they are encouraged to begin sooner rather than later. Tapering typically occurs over four weeks but can take longer to meet the needs of individual patients. The website gives the switching / tapering schedule appropriate to the patient’s current drug and dosage and can also transfer that schedule to a range of commonly used calendar programs. Where necessary, tapering schedules for other antidepressants can be entered into the website by the study team / GP. The study pharmacist will engage community pharmacists in the preparation of the tapering packages and provision of support and guidance to re-enforce the messages provided by the online intervention and patient’s GP.

- *Share:* Patients can generate a PDF file of their personalized action plan, refer to it and print out. They are encouraged to share their plan with supportive friends and family and can generate an email from the website to assist with this. They are also advised that their plan will be sent to their GP, so the GP can review and refine it and support them through the process. The patient is also scheduled to see their GP to receive updated prescriptions for their personal tapering schedule.
- *Arrange* follow-up: To support the patient through the deprescribing process and encourage adherence to their tapering plan, they receive regular automated texts with reminders to (for example) decrease dose or complete a symptom check.

A wellbeing plan for deprescribing which includes a personalized daily symptom tracker developed from the participants own data to monitor how they are feeling over the course of the tapering period. The symptom check includes monitoring psychological distress and discontinuation symptoms; high scores trigger a risk assessment (outlined below). The patient can view a graph of their scores over time and send it to their GP if they wish to.

Treating Tobacco Use and Dependence: 2008 Update U.S. Public Health Service Clinical Practice Guideline Executive Summary. Fiore MC, et al. 2008.

See Also: Park ER, Gareen IF, Japuntich S, et al. Primary Care Provider-Delivered Smoking Cessation Interventions and Smoking Cessation Among Participants in the National Lung Screening Trial. *JAMA Intern Med.* 2015;175(9):1509–1516. doi:10.1001/jamainternmed.2015.2391
