# Supplementary material for: Structured Online Support to Inform and Assist Antidepressant Deprescribing in Primary Care (WiserAD Tool): Protocol for a Pragmatic Randomized Controlled Trial
Source: JMIR Res Protoc. 2026 Aug 7;15:e81858. doi: 10.2196/81858 (PMC13449978; doi:10.2196/81858)
Supplement: Supplementary file 6 [file resprot-v15-e81858-s003.docx]

## Multimedia Appendix 3: Screenshots of the WiserAD IST
